# Identification of a Ribose-Phosphate Pyrophosphokinase that Can Interact In Vivo with the Anaphase Promoting Complex/Cyclosome

**DOI:** 10.3390/ijms18040617

**Published:** 2017-03-30

**Authors:** Haiyang Yu, Yu Zhang, Dong Zhang, Yanxi Lu, Haixia He, Fucong Zheng, Meng Wang

**Affiliations:** Hainan Key Laboratory for Sustainable Utilization of Tropical Bioresource, Institute of Tropical Agriculture and Forestry, Hainan University, Haikou 570228, China; yuhaiyang@hainu.edu.cn (H.Y.); zhangdong19921@hotmail.com (D.Z.); luyanxi3@outlook.com (Y.L.); hhxn1991@hotmail.com (H.H.); zfucong@outlook.com (F.Z.)

**Keywords:** ribose-phosphate pyrophosphokinase, 5-phospho-d-ribosyl-1-diphosphate, anaphase promoting complex/cyclosome, rubber tree

## Abstract

5-Phospho-d-ribosyl-1-diphosphate (PRPP) synthase (PRS) catalyzes the biosynthesis of PRPP, which is an important compound of metabolism in most organisms. However, no *PRS* genes have been cloned, let alone studied for their biological function in rubber tree. In this study, we identify a novel protein (PRS4) that interacts in vivo with rubber tree anaphase promoting complex/cyclosome (APC/C) subunit 10 (HbAPC10) by yeast two-hybrid assays. *PRS4* has been cloned from rubber tree and named as *HbPRS4*. Blastp search in the genome of *Arabidopsis thaliana* showed that HbPRS4 shared the highest similarity with AtPRS4, with 80.71% identity. qRT-PCR was used to determine the expression of *HbPRS4* in different tissues and under various treatments. *HbPRS4* was preferentially expressed in the bark. Moreover, the expression level of *HbPRS4* was significantly induced by the proteasome inhibitor MG132 treatment, suggesting it might be regulated by the ubiquitin/26S proteasome pathway. The amount of *HbPRS4* transcript was obviously decreased after mechanical wounding and abscisic acid (ABA) treatments, while a slight increase was observed at 24 h after ABA treatment. *HbPRS4* transcript in the latex was significantly upregulated by ethephon (ET) and methyl jasmonate (MeJA) treatments. These results suggested that HbPRS4 may be a specific substrate of HbAPC10 indirectly regulating natural rubber biosynthesis in rubber tree.

## 1. Introduction

5-phospho-d-ribosyl-1-diphosphate (PRPP) is an important compound of the metabolism in most living cells. PRPP is a precursor to synthesize pyrimidine, purine, pyridine nucleotides, tryptophan and histidine [1]. The biosynthesis of PRPP is catalyzed by PRPP synthase (PRS; EC 2.7.6.1): ribose 5-phosphate + ATP → PRPP + AMP [2]. Based on the biochemical characteristics of PRS, they can be divided into three classes. Class I PRSs are present in all living cells and require Mg^2+^ and phosphate (P) for activity, can be inhibited allosterically by ADP and prefer ATP or dATP as the diphosphoryl donor [3]. Class II PRSs are plant-specific proteins and do not depend on P for activity, lack the allosteric regulation of ADP and have a much broader specificity for the diphosphoryl donor [4,5]. Class III PRS was isolated from *Methanocaldococcus jannaschii*, which was activated by P and used only ATP or dATP as diphosphoryl donors, but lacking allosteric inhibition [6]. PRS is essential for all free living organisms, and at least one *PRS* gene has been identified in their genomes. In plant *Arabidopsis thaliana* and yeast *Saccharomyces cerevisiae*, five *PRS* genes are present in their genomes [7,8]. There are two and three *PRS* genes present in the mammals rat (*Rattus norvegicus*) and human (*Homo sapiens*), respectively [9,10,11]. At least four *PRS* (*SoPRS1–4*) are present in spinach (*Spinacia oleracea*) [5]. SoPRS1, SoPRS2, *Arabidopsis* AtPRS1 and AtPRS2 belong to Class I and require P for activity, whereas SoPRS3, SoPRS4, AtPRS3 and AtPRS4 belong to Class II and do not require P for activity [4,5,7]. Further study revealed that expressions of *AtPRS2* and *AtPRS3* were differentially regulated under P starvation. The *AtPRS2* transcript was increased in roots and shoots of P-starved plants, whereas *AtPRS3* was constitutively expressed. AtPRS3 may play a novel role in providing PRPP to cellular metabolism under P starvation [12]. These studies demonstrated that plant PRSs have a variety of functions, which are different from the bacteria and mammals’ PRSs.

Rubber tree (*Hevea brasiliensis* Müll. Arg.) is the most important source of natural rubber (NR). NR is extracted from latex of rubber tree, which is collected by cutting in the bark of the tree (called tapping). NR molecules are produced, aggregated and packaged in the laticifers of rubber tree. The latex, a cytoplasmic component of the laticifers, expels from the laticifers upon tapping. Most of the latex volume (up to 50%) is made up of rubber particles (*cis*-l,4-polyisoprene) and lutoids (20%) [13]. Latex flow and regeneration capacity after tapping are two important factors determining NR yield [14]. Natural rubber is a secondary metabolite in rubber tree, and its biosynthesis is affected by various plant hormones. Ethephon (ET, an ethylene generator) is widely used to treat the tapped bark to stimulate the latex production in rubber tree [14,15]. Jasmonic acid (JA) is another important hormone affecting latex yield, which could induce laticifer differentiation from cambium in rubber tree [16]. The content of the adenylate pool (including AMP, ADP and ATP) in the latex was increased after ET induction [17]. NR biosynthesis is a process consuming energy. Energy availability, mainly constituted with the total content of adenine nucleotide, is a major factor affecting NR regeneration between two tappings [14]; while PRPP is a precursor to synthesize adenine nucleotide [1]. In earlier research, PRS preparations have been purified from rubber tree latex, which depend on Mg^2+^ and P for activity, and used only ATP as the diphosphate group donor [18]. However, so far, no *PRS* genes have been cloned, let alone studied for their biological functions in rubber tree.

The anaphase promoting complex/cyclosome (APC/C) is a multi-subunit E3 ubiquitin ligase that regulates cell-cycle progression by ubiquitinating and degrading mitotic regulatory proteins via ubiquitin/26S proteasome system [19,20]. Since the APC/C plays a key role in regulating cell-cycle, it has been well studied in model species, such as yeast and *Arabidopsis*. The APC/C is constituted by 11–13 subunits [19,21]. APC10/destruction of cyclin B protein 1 (Doc1) plays a direct and critical role in recognizing substrates [22,23]. The increase of APC10 expression in yeast can enhance the life span [24]. AtAPC10 regulates the leaf and vascular development in *Arabidopsis* [25,26]. In rice (*Oryza sativa* L.), the interaction of OsTAD1 and OsMOC1, together with OsAPC10 forms a complex and regulates rice tillering [27,28]. These studies suggested that APC/C played multiple functions by regulating the ubiquitination of specific substrates. Although the crucial role of APC/C has been characterized in model species, limited study in tree species has been reported. In this study, we identify a novel protein (ribose-phosphate pyrophosphokinase 4, PRS4) that interacts in vivo with the *Hevea brasiliensis* APC/C subunit 10 (HbAPC10) using the yeast two-hybrid system. *PRS4* cloned from *Hevea brasiliensis* was named as *HbPRS4*. qRT-PCR (quantitative real-time PCR) was used to determine the expression of *HbPRS4* in different tissues and under different treatments. The results in this study suggested that HbPRS4 may serve as a specific substrate of HbAPC10 indirectly regulating NR biosynthesis in rubber tree.

## 2. Results

### 2.1. Identification and Isolation of Rubber Tree PRS4

**Figure 1:**
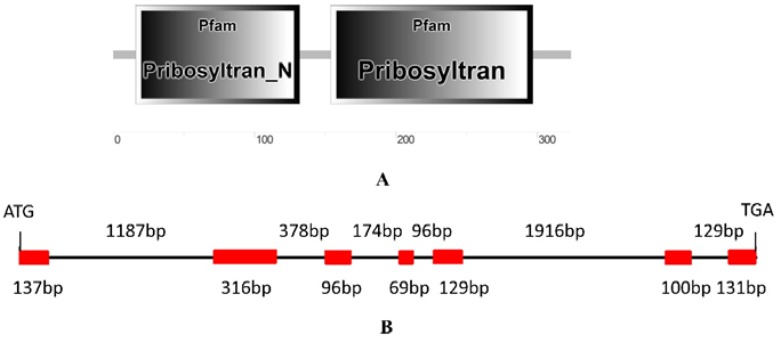
Protein architecture of HbPRS4 (**A**) and its encoding gene structure (**B**). (**A**) The protein architecture of HbPRS4 was predicted using the SMART online service; (**B**) six introns are shown by lines, and their corresponding length is indicated above the lines; and seven exons are shown by boxes, and their corresponding length is indicated below the boxes.

A normalized cDNA library derived from the latex of rubber tree clone CATAS 7-33-97 (bred by Chinese Academy of Tropical Agricultural Sciences) was screened using the yeast two-hybrid system, and a positive clone showed a strong interaction with the bait pGBKT7-HbAPC10. Then, this clone was isolated and sequenced. It did not contain a full-length of the target gene since it was shorter than its homologous genes. The sequence of this positive clone was used for blastn searching in the transcriptome shotgun assembly (TSA) database of rubber tree. A TSA sequence (GenBank accession: JT933457) obtained from rubber tree clone RRIM 600 contained the ORF of target gene, and then, the full length of the ORF was amplified by RT-PCR in CATAS 7-33-97 and sequenced. The amplified cDNA has a complete ORF that is predicted to encode a protein of 325 amino acids with a molecular mass of 36.3 kDa and a theoretical isoelectric point of 6.89. Since blastp search in the genome of *Arabidopsis thaliana* showed that this putative protein shared the highest similarity with AtPRS4 (with 80.71% identity), it was designated as HbPRS4. Protein architecture analysis by NCBI conserved domain search and SMART online service demonstrated that HbPRS4 has a Pribosyltran_N domain (from the 16th–132nd amino acid) and a Pribosyltran domain (from the 154th–297th amino acid) (Figure 1A).

**Figure 2:**
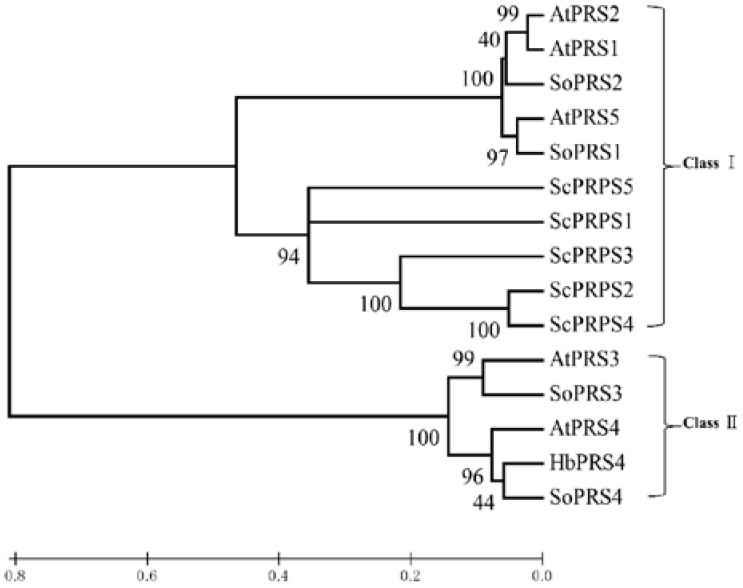
Phylogenetic tree of HbPRS4 and other PRS (5-phospho-d-ribosyl-1-diphosphate synthase) proteins. The sequences of all of the PRS proteins were aligned using ClustalX 1.83. A phylogenetic tree was generated based on the neighbor-joining method in MEGA 6.06. Bootstrap values (for 1000 replicates) are shown at the nodes. GenBank accessions of the proteins used in this study are as follows: *Arabidopsis thaliana* AtPRS1–AtPRS5 (CAA58717, CAA63552, CAB43552, CAB43553, At2g44530); *Spinacia oleracea* SoPRS1–SoPRS4 (CAB43599, CAB43600, CAB43601, CAB43602); *Saccharomyces cerevisiae* ScPRPS1–ScPRPS5 (CAA49674, CAA52436, CAA52437, CAA84888, CAA62523).

Blastn searching in the genome database derived from CATAS 7-33-97 indicated that there was a scaffold containing the full length of *HbPRS4* (GenBank accession: LVXX01000028). The genomic sequence of *HbPRS4* was extracted and used for further analysis of the gene structure. Comparing the genomic sequence of *HbPRS4* with its corresponding cDNA sequence, it was revealed that there were seven exons and six introns present in the genomic sequence of *HbPRS4*, with the total length of 4858 bp (Figure 1B). Phylogenetic analysis indicated that HbPRS4 showed a high degree of similarity with *Spinacia oleracea* SoPRS4 and *Arabidopsis thaliana* AtPRS4. HbPRS4, together with SoPRS3, SoPRS4, AtPRS3 and AtPRS4 belonged to Class II, while plant AtPRS1, AtPRS2, AtPRS5, SoPRS1, SoPRS2 and yeast ScPRPS1-ScPRPS5 gathered into another branch and belonged to Class I (Figure 2).

### 2.2. Confirmation of the Interaction of HbAPC10 with HbPRS4 by Yeast Two-Hybrid Assays

**Figure 3:**
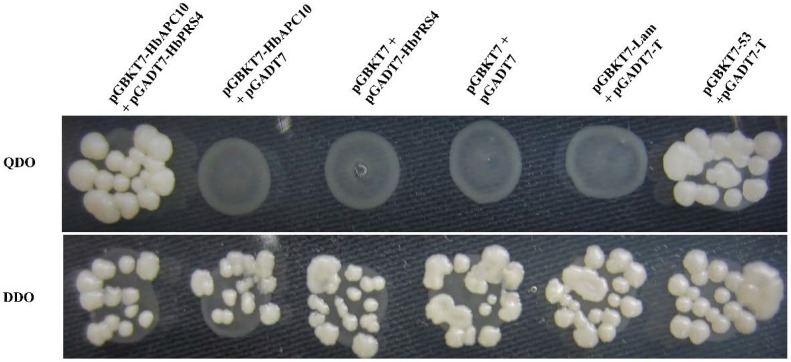
Yeast two-hybrid assays tested the interaction of HbAPC10 with HbPRS4. Transformed yeast cells were grown on synthetic dropout (SD) nutrient mediums DDO (SD/-Leu/-Trp) and QDO (SD/-Ade/-His/-Leu/-Trp). pGBKT7-53 with pGADT7-T co-transformation was used as a positive control. pGBKT7-Lam with pGADT7-T, pGBKT7-HbAPC10 with pGADT7, pGBKT7 with pGADT7-HbPRS4, and pGBKT7 with pGADT7 co-transformations were used as negative controls.

In order to confirm the interaction of HbAPC10 with HbPRS4, the ORF of *HbPRS4* was cloned into the pGADT7 vector to construct a prey plasmid pGADT7-HbPRS4 and used for yeast two-hybrid assays. The prey plasmid pGADT7-HbPRS4 and the bait plasmid pGBKT7-HbAPC10 were co-transformed into yeast strain AH109 and grown on synthetic dropout (SD) nutrient mediums DDO (SD/-Leu/-Trp) and QDO (SD/-Ade/-His/-Leu/-Trp) for the interaction test. Yeast two-hybrid assays revealed that there was no interactions in a series of negative controls, i.e., pGBKT7-HbAPC10 with pGADT7, pGBKT7 with pGADT7-HbPRS4, pGBKT7 with pGADT7 and pGBKT7-Lam with pGADT7-T. However, pGADT7-HbPRS4 interacted strongly with pGBKT7-HbAPC10, and a strong interaction was also observed in the positive control (pGADT7-53 with pGBKT7-T) (Figure 3). This result demonstrated that HbPRS4 interacts in vivo with HbAPC10.

### 2.3. Transcriptional Profiles of HbPRS4 in Different Tissues and in Response to Different Stimulus

**Figure 4:**
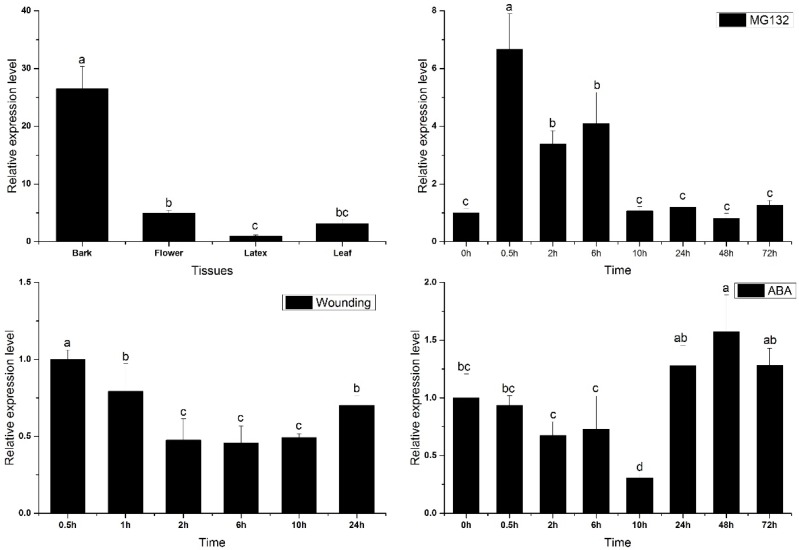
*HbPRS4* expression in different tissues and under different treatments. For different tissues, analysis was performed in tapping trees. Mechanical wounding, MG132 and ABA (abscisic acid) treatments were performed in seedlings. Different lowercase letters above the bars show significant differences at *p* < 0.05 (probability level) by one-way ANOVA with Tukey’s test.

**Figure 5:**
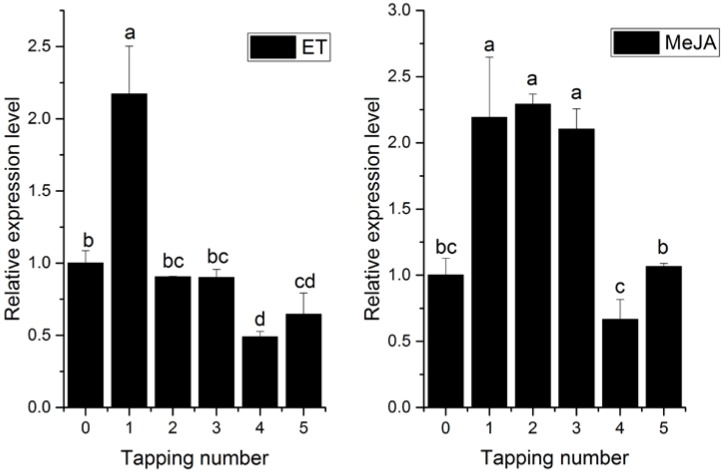
*HbPRS4* expression in response to ET (ethephon) and MeJA (methyl jasmonate) stimulus. One-point-five percent (*v*/*v*) ET and 1.0% (*v*/*v*) MeJA were used to treat the tapping cut on the bark of tapping trees. Different lowercase letters above the bars show significant differences at *p* < 0.05 (probability level) by one-way ANOVA with Tukey’s test.

As shown in Figure 4, *HbPRS4* was constitutively expressed in rubber tree, but its expression level was varied between different tissues. The maximum was present in the bark, flower and leaf with moderate expression, which was 26.5-, 5- and 3.1-fold over that in the latex. The preferential expression of *HbPRS4* in the bark indicated its main function in this tissue. Yeast two-hybrid assays demonstrated that HbPRS4 can interact with HbAPC10, and APC10 is an E3 ubiquitin ligase regulating protein degradation via the ubiquitin/26S proteasome pathway. Therefore, MG132, an inhibitor of the 26S proteasome, was used to treat rubber tree seedlings of CATAS 7-33-97. The *HbPRS4* transcript was rapidly induced by MG132. It reached the largest amount at 0.5 h and increased 6.7-fold compared with the control (0 h) (Figure 4). This suggested that *HbPRS4* was regulated by the ubiquitin/26S proteasome pathway. To systematically study the expression profiles of *HbPRS4*, the effect of several plant hormones and mechanical wounding on the *HbPRS4* transcript were tested in both tapping trees and seedlings of CATAS 7-33-97. After mechanical wounding treatment, the *HbPRS4* transcript was continuously decreased, and it reduced by 55% at 6 h compared with 0 h. After abscisic acid (ABA) treatment, the amount of *HbPRS4* transcript was obviously decreased and reduced by 70% at 10 h compared with 0 h, but there was a slight increase from 24 h (Figure 4). Ethephon (ET) and methyl jasmonate (MeJA) treatments were performed in tapping trees, then the latex was extracted and used for expression analysis after treatments. Application of exogenous ET and MeJA in the bark can significantly increase *HbPRS4* expression (Figure 5). As for the ET treatment, the highest expression occurred at the first tapping, and then, it decreased slightly from the second tapping (Figure 5). *HbPRS4* expression was increased more than two-fold at the first to third tapping after MeJA treatment, then it returned to the untreated control level at the fourth tapping (Figure 5). These results suggested that *HbPRS4* was regulated by multiple hormones.

## 3. Discussion

PRS catalyzes the biosynthesis of PRPP, which plays a critical role in nucleotide biosynthesis, the salvaging of nucleobases and tryptophan and histidine synthesis [1]. The functions of the PRSs have been investigated systematically in *Saccharomyces cerevisiae*. The ScPRS interacts with each other and exists in vivo as three minimal functional entities, ScPRS1/ScPRS3, ScPRS2/ScPRS5 and ScPRS4/ScPRS5. Each of the multimeric complexes can support yeast viability [29,30]. Moreover, ScPRS1 and ScPRS5 interact with the cell wall integrity pathway [30,31]. In *Arabidopsis* and tobacco plants, overexpression of the *Ashbya gossypii AgPRS2,4* gene or its mutated variant could increases PRS activity and substantially enhances biomass accumulation along with significant changes in the content of sugars and other metabolites [32]. The bark of rubber tree is regularly tapped for harvesting latex. Latex is a cytoplasmic component of laticifers [13], containing the cytosol, vacuoles (the lutoids), plastids, mitochondria, nuclei and endoplasmic reticulum [33,34]. It also contains polysomes and numerous rubber particles [35]. Upon tapping, most cytoplasmic components are expelled from the laticifers, while nuclei and mitochondria remain adhered to the plasmalemma for latex regeneration after tapping. Therefore, sufficient availability of PRPP is essential for latex regeneration in rubber tree, which is an important metabolite for nucleotide and protein biosynthesis. These suggested that PRSs of rubber tree are crucial to latex regeneration, especially nucleotide and protein biosynthesis after tapping. In a previous study, PRS preparations had been purified from rubber tree latex. Biochemical analysis revealed that Mg^2+^ and P affected the activity of this latex PRS; and it used only ATP as the diphosphate group donor [18]. Based on the biochemical characteristics of this latex PRS and classification of PRS family proteins, it is safe to deduce that this latex PRS belongs to Class I PRS. In this study, phylogenetic analysis supported HbPRS4 as a Class II PRS with a high degree of similarity with *Spinacia oleracea* SoPRS4 and *Arabidopsis thaliana* AtPRS4 (Figure 2), which is different from the previous PRS purified from rubber tree latex in Gallois et al.’s study [18].

Comparing the expression of *HbPRS4* in different tissues of rubber tree revealed that it was preferentially expressed in the bark (Figure 4). This result suggested its function mainly in the bark. Furthermore, the *HbPRS4* transcript was decreased after mechanical wounding and ABA treatments (Figure 4). However, application of exogenous ET and MeJA in the bark can significantly increase *HbPRS4* expression (Figure 5). ET is widely used in natural rubber production, which can effectively increase latex production in rubber tree by delaying coagulation and prolonging latex flow in laticifers [35]. Studies on the physiological changes of latex in response to ET stimulation in the bark of rubber tree indicated that the amount of adenine nucleotides (especially ATP and ADP) in the latex was significantly increased after 13–21 h of treatment [17]. The increase of energy supplies should be prior to material synthesis during latex regeneration, since ATP is required for most catalytic reactions, and PRS is no exception. From this point, it is easy to understand that the increase of *HbPRS4* expression (occurring at 72 h after ET treatment) in this study was later than the increase of total adenine nucleotides in Amalou et al.’s study [17]. Blastn searching in the rubber tree genome database indicated that there was another scaffold (GenBank accession: LVXX01000564) sharing 84% identity with *HbPRS4*. Together with the latex PRS purified by Gallois et al. [18], we speculated that there was more than one *PRS* gene present in the rubber tree genome. These PRSs may work together in response to ET induction. The effect of ET on PRS activity needs to be further studied, since it controls the synthesis of PRPP, which is a requirement for nucleotide and protein biosynthesis associated with latex regeneration.

APC10 is an E3 ubiquitin ligase and plays a direct and crucial role in ubiquitinating specific substrates, which will be degraded by the 26S proteasome [20,22,23]. In rice, OsAPC10 interacts with the OsMOC1-OsTAD1 complex and regulates rice tillering [27,28]. Overexpression of AtAPC10 in *Arabidopsis* accelerates CYCLIN B1;1 (CYCB1;1) degradation and cell division at the early stages of leaf development and leads to increasing final cell number and leaf size [25,26]. APC10 may take part in regulating multiple physiological processes by ubiquitinating specific substrates. In this study, we identified a novel protein HbPRS4, which can interact in vivo with rubber tree HbAPC10 (Figure 3). Since PRPP is the catalytic reaction product of PRS, the interaction of HbAPC10 with HbPRS4 might regulate the dynamic balance of PRPP in the latex, which might indirectly regulate latex regeneration and/ or laticifer division in the bark of rubber tree. While no more of the PRPP was needed in the latex, HbAPC10 may ubiquitinate HbPRS4 for degradation. However, the relationship between PRPP content with the HbAPC10-HbPRS4 complex in the latex of rubber tree needs to be studied. These results in this study provide a new line for studying the regulation of natural rubber production in rubber tree.

## 4. Materials and Methods

### 4.1. Plant Materials and Treatments

Rubber tree clone CATAS 7-33-97 (bred from crossing RRIM 600 × PR 107) was planted at the experimental field of Institute of Tropical Agriculture and Forestry, Hainan University, Danzhou City, China. Grafted budding seedlings of CATAS 7-33-97 were used for wounding, 50-µmol·L^−1^ MG132 (an inhibitor of proteasome) and 200-µmol·L^−1^ ABA treatments. These treatments were performed according to our previous report [36]. ET and MeJA treatments and different tissues’ analysis were performed in tapping trees of CATAS 7-33-97. One-point-five percent (*v*/*v*) ET and 1.0% (*v*/*v*) MeJA were spread over the tapping cut on the bark, and 3 trees were used for each treatment. The trees were tapped every three days after treatments, and latex was collected for total RNA extraction.

### 4.2. Yeast Two-Hybrid Screening

The *Arabidopsis* AtAPC10 (GenBank accession: NP_565433) sequence was used for tblastn searching in the *Hevea brasiliensis* TSA database, and an accession (JT940912) from RRIM 600 encoding a completed ORF of rubber tree APC10 (*HbAPC10*) was obtained. In order to screen the interacting protein of HbAPC10, the ORF of *HbAPC10* was amplified by RT-PCR in CATAS 7-33-97 and then cloned into the EcoRI/BamHI restriction sites of pGBKT7 to generate the bait vector pGBKT7-HbAPC10. This bait vector was transformed into the yeast strain Y2HGold to generate a bait clone. A normalized yeast two-hybrid cDNA library derived from the latex of CATAS 7-33-97 cloned in the pGADT7 vector was introduced into yeast strain Y187 using the Make Your Own “Mate & Plate™” Library System (Clontech, Mountain View, CA, USA) [37]. The bait clone pGBKT7-HbAPC10 in Y2HGold was used for yeast two-hybrid screening according to the recommended protocol of the Matchmaker™ Gold Yeast Two-Hybrid System (Clontech). The bait clone was mated with the prey library by incubation at 30 °C for 24 h, and positive interactions were selected on QDO medium (SD/-Ade/-His/-Leu/-Trp) at 30 °C for 5 days. The positive interactions were tested on QDO medium 2 times. Interacting cDNA clones were isolated and transformed into *Escherichia coli* strain DH5α and then sequenced with the T7 promoter primer to determine the sequence of the inserted cDNA.

To analyze the interaction between HbAPC10 and HbPRS4, the ORF of *HbPRS4* was amplified and cloned into pGADT7 to construct a prey plasmid pGADT7-HbPRS4, and pGBKT7-HbAPC10 was used as the bait plasmid. Primers used for generating constructs for yeast two-hybrid assays are listed in Table 1. Since the interaction of p53 with large T-antigen has been confirmed by the yeast two-hybrid system [38,39], pGBKT7-53 (which encodes the Gal4 DNA-BD fused with murine p53) with pGADT7-T (which encodes the Gal4 AD fused with SV40 large T-antigen) co-transformation was used as a positive control. pGBKT7-Lam (which encodes the Gal4 BD fused with lamin) and pGADT7-T was used as a negative control. pGBKT7-HbAPC10 with pGADT7, pGBKT7 with pGADT7-HbPRS4, and pGBKT7 with pGADT7 were also used as negative controls. Yeast strain AH109 was co-transformed with specific bait and prey constructs. All yeast transformants were cultivated on DDO (SD/-Leu/-Trp) and QDO mediums for the interaction test.

### 4.3. Isolation of HbPRS4 and Bioinformatic Analysis

The partial sequence of *HbPRS4* was obtained by sequencing the positive clone of the yeast two-hybrid screening HbAPC10 interacting proteins. The partial sequence of *HbPRS4* was performed blastn searching in the *Hevea brasiliensis* TSA database. A sequence (GenBank accession: JT933457) generated from rubber tree RRIM 600 contained the whole ORF of *HbPRS4*. Primers PRS4-F1 and PRS4-R1 (Table 1) were used to amplify *HbPRS4* by RT-PCR from CATAS 7-33-97. The amplified product was cloned into the pGEM-T Easy vector (Promega, Madison, WI, USA) and sequenced.

The theoretical isoelectric point and molecular weight of HbPRS4 was calculated by the ProtParam tool (http://web.expasy.org/compute_pi/). NCBI conserved domain search (http://www.ncbi.nlm.nih.gov/Structure/cdd/wrpsb.cgi?) and SMART (http://smart.embl-heidelberg.de/) were used to identify the architecture of HbPRS4. Sequences of all the PRS proteins were aligned by the ClustalX 1.83 [40]. The phylogenetic tree was generated by MEGA Version 6.06 based on the neighbor-joining method with a Poisson correction model and a bootstrap test of 1000 replicates [41].

### 4.4. RNA Extraction and qRT-PCR Analysis

Total RNA was extracted with the RNAprep Pure Plant Kit (Polysaccharides & Polyphenolics-rich) (TIANGEN, Beijing, China) and used for cDNA synthesis with the RevertAid^TM^ First Strand cDNA Synthesis Kit (Fermentas, Burlington, ON, Canada) according to the recommended protocol. Specific primers designed for *HbPRS4* (PRS4-QF3 and PRS4-QR3, Table 1) were used for qRT-PCR amplification, and *HbActin* (GenBank accession: HO004792, Actin-F and Actin-R, Table 1) was used as the housekeeping gene. The PCR reaction was performed according to our previous report [36]. The 2^−ΔΔ*CT*^ (cycle threshold) algorithm was used to calculate the relative expression level of target gene [42]. For each sample, qRT-PCR was performed with three technical replicates and three biological replicates. The data were analyzed with IBM-SPSS 23.0. The data for replicates were analyzed by one-way ANOVA, and multiple comparisons were performed by the Tukey test at *p* < 0.05. Figures were drawn and viewed by OriginPro 9.0.

## 5. Conclusions

Yeast two-hybrid assays demonstrated that HbPRS4 can interact in vivo with HbAPC10. This is the first report on the interaction of APC10 with PRS4, and *HbPRS4* gene was first cloned from rubber tree. Phylogenetic analysis supported HbPRS4 as a Class II PRS with a high degree of similarity with *Spinacia oleracea* SoPRS4 and *Arabidopsis thaliana* AtPRS4. *HbPRS4* was preferentially expressed in the bark. Moreover, the expression level of *HbPRS4* was significantly induced by the proteasome inhibitor MG132 treatment, suggesting that *HbPRS4* was regulated by the ubiquitin/26S proteasome pathway. *HbPRS4* transcripts in the latex were remarkably upregulated by ET and MeJA stimulus. These results suggested that HbPRS4 may be a specific substrate of HbAPC10 indirectly regulating natural rubber biosynthesis in rubber tree.

## Figures and Tables

**Table 1:** Sequences of the primers used in this study.

Name	Sequence (5′ to 3′)	Name	Sequence (5′ to 3′)
PRS4-F1	GGGACTCTCCTTCCAGCTCT	PRS4-R1	CGTGAAACACCAATGGACAA
APC10-EcoRI-F	CGGAATTCATGGCAACAGAGTCAT	APC10-BamHI-R	CGGGATCCTCATCTCACTGAAGAGT
PRS4-EcoRI-F2	CGGAATTCATGGAAAAGAAGGCAGA	PRS4-XhoI-R2	CCGCTCGAGTCATATTTGCAGAGCA
PRS4-QF3	CCCTCTGCTAAAGCAACGTC	PRS4-QR3	CCATGGGAAAATGATCCAAC
Actin-F	GATGTGGATATCAGGAAGG	Actin-R	CATACTGCTTGGAGCAAGA

The underlined sequence indicated the restriction enzyme cutting site.

## Abbreviations

PRS4	Ribose-phosphate pyrophosphokinase 4
PRPP	5-Phospho-d-ribosyl-1-diphosphate
APC/C	Anaphase promoting complex/cyclosome
ET	Ethephon
MeJA	Methyl jasmonate
ABA	Abscisic acid
qRT-PCR	Real-time quantitative PCR
ORF	Open reading frame
TSA	Transcriptome shotgun assembly
QDO	SD/-Ade/-His/-Leu/-Trp
DDO	SD/-Leu/-Trp
PCR	Polymerase chain reaction
RT-PCR	Reverse transcription PCR
qRT-PCR	Quantitative real time PCR
ATP	Adenosine triphosphate
ADP	Adenosine diphosphate
AMP	Adenosine monophosphate
CT	Cycle threshold
